# What's new in sports cardiology? Hot topics from “Sport and Heart,” First International Congress in Sports Cardiology

**DOI:** 10.1002/clc.24034

**Published:** 2023-05-25

**Authors:** Liuba Fusco, Luigi Sciarra

**Affiliations:** ^1^ Cardiology Department New University Hospital Northampton Northampton UK; ^2^ Department of Cardiovascular Diseases University of L'Aquila L'Aquila Italy

Between 17 and 19 November 2022, under the auspices of the Italian Society of Sports Cardiology (SIC Sport), the first international congress of SIC sport entitled “Sport and Heart ‐ From science to clinical practice ‐ First International Congress on sport cardiology” was held in Rome. The conference was held under the direction of the Chairman Professor Luigi Sciarra, president of the SIC Sport. The congress offered a unique opportunity to delve deeper into the interrelationship between sport and cardiology issues by bringing together leading academics, experienced clinicians, and marketing industry experts. Given the growing interest in sport cardiology, this special issue wants to highlight some of the main topics covered over the course of the 3 days. Even though the first records of academic interest toward physical activity date back to the fifth century BC, with the father of sport medicine Herodicus, it wasn't until the 1970s that sport cardiology developed into a specialty of its own.^1^ Since then, sport medicine has made leaps and bounds and now, with the recent digital health revolution, our approach to athletes is once again taking on new forms.

The International Sport Cardiology Congress covered a wide range of new challenges currently being faced by the medical community, such as how to gather the stock of existing cardiovascular preparticipation screening (PPS) and the integrated policy approaches that have been put into practice in Italy and throughout Europe.

Professor Christopher Cannon, from Harvard Medical School, opened the Congress with his magisterial lecture on “Cardiovascular risk and physical activity in the Third Millennium”; providing the modern concept of physical exercise as an appropriate therapy that should be prescribed to reduce cardiovascular risk in many clinical settings. In healthy population groups, moderate to vigorous physical activity was associated with longer life expectancy,^2^ thanks to its pleiotropic effects.^3^ Furthermore, Prof Cannon showed the well‐established paradigm shift regarding the efficacy and safety of exercise in patients with ischemic diseases^4^ or chronic heart failure,^5^ a favorable effect extended even to moderate intensity physical activity.^6^

Going straight to the heart of the conference, challenging clinical scenarios in athletes were discussed as congenital heart diseases or acquired cardiac diseases and the possibilities and risks concerning competitive sports participation. The invited speakers suggested replacing the binary yes‐or‐no eligibility in sports with a more nuanced approach, using a large amount of data from the studies. This emerging updated information demonstrates the risk of some cardiac conditions seems to be lower than previously projected. Fundamentally, it appears mandatory to reduce the risk of sudden cardiac death (SCD) while simultaneously avoiding unnecessary sports restrictions. For these reasons, the PPS for cardiovascular diseases carried out before any physical activity is crucial and in this field, Italy has shown up to have a leading history, since 1982 until our days.^7^ As evidenced by the study of Prof Sarto et al., the PPS, with appropriate risk stratification for SCD and related clinical treatment, can help to reduce mortality during long‐term follow‐up.^8^ The screening for return‐to‐play has also gained importance in the last period, and its cost for the national health systems was addressed during the conference, as well.^9^, ^10^, ^11^

An entire session was dedicated to discussing the European Union Recovery and Resilience Plan: a wide‐ranging response aimed at mitigating the economic and social impact of the coronavirus pandemic and making European economies and societies better geared to future challenges. During this session, a cross‐country analysis highlighted a range of interrelated areas such as “Investing in digital health literacy” to assess Health Resilience.

The conference also dealt with topics like telemedicine, which has become an integral component of everyday clinical practice for patients with cardiovascular diseases, as well as rapid advances in wearable technologies and real‐time monitoring, which offer practical applications for medical and preventive support in dealing with athletes' care. Medical technology industry representatives at the conference highlighted the importance of the latter, wearables and real‐time monitoring, as the key areas for synergies between practitioners and Med‐Tech.

In conclusion, sport cardiology is a rapidly evolving discipline. This congress has represented a small step forward toward a better understanding of how recommendations are likely to change at an increasingly fast pace, too. The time has come to adopt the notion of individualized medicine in sport cardiology. One that promotes safety, without unnecessary limitations for the athlete, through a patient‐centered approach and shared decision‐making.
